# Strains of *Anaplasma phagocytophilum* from horses in Ohio are related to isolates from humans in the northeastern USA

**DOI:** 10.1128/spectrum.02632-23

**Published:** 2023-10-26

**Authors:** Rory C. Chien, Lin Mingqun, Qi Yan, Nina Randolph, Weiyan Huang, Maxey Wellman, Ramiro Toribio, Yasuko Rikihisa

**Affiliations:** 1 Laboratory of Molecular, Cellular, and Environmental Rickettsiology, Infectious Diseases Institute, The Ohio State University, Columbus, Ohio, USA; 2 Department of Veterinary Biosciences, College of Veterinary Medicine, The Ohio State University, Columbus, Ohio, USA; 3 Department of Veterinary Clinical Sciences, College of Veterinary Medicine, The Ohio State University, Columbus, Ohio, USA; UJF-Grenoble 1, CHU Grenoble, Grenoble, France

**Keywords:** *Anaplasma phagocytophilum*, horses, *ankA*, *p44*, equine granulocytic anaplasmosis

## Abstract

The tick-borne obligatory intracellular bacterium *Anaplasma phagocytophilum* infects granulocytes of humans as well as domesticated and wild animals, causing a febrile disease collectively called granulocytic anaplasmosis. The host species specificity and zoonotic potential of *A. phagocytophilum* strains remain unclear. In the USA, human granulocytic anaplasmosis (HGA) is primarily reported in Wisconsin and Minnesota, the northeastern coastal states, and northern California. In horses, *A. phagocytophilum* causes equine granulocytic anaplasmosis (EGA). Although EGA has been reported in the same US regions as HGA, horses with a definitive diagnosis of EGA have not been reported in Ohio, where the incidence of HGA is low. In this study, samples from archived blood smears or whole blood from three horses from three different counties in Ohio that presented in 2018, 2020, and 2021 with clinical signs and laboratory findings consistent with EGA were evaluated by PCR and sequencing. Sequences of *ankA* (encoding ankyrin A) of the three *A. phagocytophilum* strains were identical and related to strains isolated from humans in the northeastern USA yet distinct from those in California or Europe based on phylogenetic analysis. Each Ohio strain contains unique *A. phagocytophilum p44* genes encoding the immunodominant major outer membrane proteins, underscoring the antigenic diversity of *A. phagocytophilum* strains that cause EGA in Ohio. From 2018 to 2023, 14 additional cases of EGA were identified by serodiagnosis in Ohio and nearby locations in Pennsylvania, pointing to a heretofore unrecognized prevalence of EGA and the risk of transmission of anaplasmosis to humans and animals in this region.

**IMPORTANCE:**

The tick-borne obligatory intracellular bacterium *Anaplasma phagocytophilum* infects humans as well as domesticated and wild animals, causing a febrile disease collectively called granulocytic anaplasmosis. The epidemiology and the host species specificity and zoonotic potential of *A. phagocytophilum* strains remain unclear. In this study, *ankA* (encoding ankyrin A) and *p44* gene sequences of *A. phagocytophilum* were determined in clinical specimens from horses in Ohio and compared with those found in *A. phagocytophilum* strains from various hosts and geographic regions. With increasing numbers of seropositive horses, the study points out the unrecognized prevalence and uncharacterized strains of *A. phagocytophilum* infection in horses and the importance of *A. phagocytophilum* molecular testing for the prevention of equine and human granulocytic anaplasmosis.

## INTRODUCTION

Arthropod-borne diseases, including human granulocytic anaplasmosis (HGA) caused by *Anaplasma phagocytophilum* infections, are on the rise, probably because of changes in both human activities and climate (1). The number of HGA cases in the USA has increased from 348 in 2000 to 6,729 in 2021, a rate that is higher than that for any other tick-borne disease (2). The cases have been confined primarily to Wisconsin, Minnesota, and the northeastern coastal states, where ticks of the genus *Ixodes* are abundant (2, 3).

*A. phagocytophilum* is a small Gram-negative pleomorphic coccus of the family *Anaplasmataceae* and order Rickettsiales (4). *A. phagocytophilum* infects granulocytes and endothelial cells, causing febrile illnesses accompanied by leukopenia, thrombocytopenia, and mild hepatitis in humans and domesticated animals, and opportunistic infections or other complications may follow (5, 6). In horses, *A. phagocytophilum* causes equine granulocytic anaplasmosis (EGA) (7). Diverse genotypes and strains of *A. phagocytophilum* have been reported globally from a variety of hosts, including ticks, humans, and wild and domesticated animals (6, 8, 9).

Neither natural *A. phagocytophilum* infection of domesticated animals, including horses, has been reported in Ohio, nor the existence of *A. phagocytophilum* strains in Ohio has been examined. Here, we report the results of an *A. phagocytophilum* strain analysis of the first molecularly confirmed cases of EGA in Ohio. Analysis of a hypervariable functional gene, namely, *ankA* (encoding ankyrin A), that is unique to the bacterium *A. phagocytophilum* revealed that these equine strains are related to isolates from HGA patients in the state of New York (10, 11). Analysis of *p44/msp2* (encoding major surface antigen P44/Msp2) showed potential antigenic diversity of *A. phagocytophilum* strains that cause EGA in Ohio. Thus, EGA may serve as a sentinel for HGA, underscoring the epidemiological and zoonotic implications of *A. phagocytophilum*, the increase in east-to-west vector migration, and the importance of monitoring *A. phagocytophilum* infection in domesticated and wild animals for the prevention of HGA.

## MATERIALS AND METHODS

### Specimens and DNA extraction

DNA was isolated from three horses that were presented to the Ohio State University Veterinary Medical Center with clinical signs and laboratory data consistent with EGA. Archived buffy-coat smears with microscopically observable bacterial morulae (microcolonies) were used for two horses (ID: BP18 and MK20). Coverslips were removed with liquid nitrogen (12). Blood cells were scraped off the slides and used for DNA extraction using Chelex 100 Resin (Bio-Rad Laboratories, Hercules, CA) as described previously (13). A blood sample collected in a tube containing EDTA (anticoagulant) was obtained from a third horse (GL21) for DNA extraction using a QIAamp DNA Blood Mini Kit (Qiagen, Valencia, CA).

### PCR

#### ankA

Nested PCR was conducted for *A. phagocytophilum ankA*. The first-step PCR was performed in 50-µL reactions containing 5–15 µL horse-blood DNA, 20 pmol each of primers *AnkA*_F and *AnkA*_R (Table S1), 0.2 mM deoxynucleotide triphosphate (dNTP; each), 2 mM MgCl_2_, 1.25 U *Taq* DNA polymerase (New England Biolabs, Ipswich, MA), and 1× Standard *Taq* Reaction Buffer (New England Biolabs) with thermal cycling as follows: denaturation at 95°C for 5 min, followed by 35 cycles of denaturation at 95°C for 10 s, annealing at 52°C for 30 s, and extension at 68°C for 1 min, with one final extension at 68°C for 5 min. The second-step PCR was performed in 25-µL reactions containing 3 µL of amplicons from the first-step *AnkA* PCR, 10 pmol each of primers *AnkA_F2* and *AnkA_R2*, 0.2 mM dNTP (each), 1 mM MgCl_2_, 0.625 U *Taq* DNA polymerase, and 1× Standard *Taq* Reaction Buffer with thermal cycling as follows: denaturation at 95°C for 5 min, followed by 35 cycles of denaturation at 95°C for 10 s, annealing at 52°C for 30 s, and extension at 68°C for 30 s, with one final extension at 68°C for 5 min.

#### p44

PCR for *A. phagocytophilum p44* was performed with 20 pmol each of primers p3708 and p4257 (Table S1) targeting a conserved region of *p44* homologs (14) in 50-µL reactions containing 5–15 µL horse-blood DNA, 0.2 mM dNTP (each), 1 U Q5 High-Fidelity DNA Polymerase (New England Biolabs), and 1× Q5 reaction buffer (New England Biolabs). PCR thermal cycling was as follows: denaturation at 98°C for 5 min, followed by 35 cycles of denaturation at 98°C for 10 s, annealing at 56°C for 30 s, and extension at 72°C for 30 s, with one final extension at 72°C for 7 min.

#### HPRT

PCR was performed in 25-µL reactions containing 2 µL horse-blood DNA, 10 pmol each of primers *HPRT1P3-F* and *HPRT1P3-R* (Table S1), 0.2 mM dNTP (each), 1.25 U *Taq* DNA polymerase, and 1× Standard *Taq* Reaction Buffer (New England Biolabs) with thermal cycling as follows: denaturation at 94°C for 5 min, followed by 35 cycles of denaturation at 94°C for 15 s, annealing at 58°C for 30 s, and extension at 68°C for 30 s, with one final extension at 68°C for 5 min.

For each PCR product, 5 µL was examined by electrophoresis through a 1.5% agarose gel containing 0.5 µg/mL ethidium bromide. Amplicons of approximately 500, 200, and 183 bp from *p44*, *ankA*, and *HPRT*, respectively, were visualized using the Amersham AI680QC gel documentation system (GE Healthcare, Marlborough, MA).

### Cloning, DNA sequencing, and analysis

The *A. phagocytophilum ankA* amplicons from the nested PCR were purified using the GeneJET PCR Purification Kit (Thermo Fisher Scientific, Waltham, MA) or QIAquick Gel Extraction Kit (Qiagen). The *p44* amplicons were purified using the GeneJET PCR Purification Kit and cloned into plasmid pCR-Blunt II-TOPO (Invitrogen, Waltham, MA). Cloned plasmids were transformed into NEB5α competent *Escherichia coli* cells (New England Biolabs) by 42°C heat shock and selected on Luria-Bertani agar plates containing 50 µg/mL kanamycin. Multiple single bacterial colonies were obtained from the selective plate of each clone, and *p44*-bearing plasmids were purified with the GeneJET Plasmid Miniprep Kit. Purified *p44*-bearing plasmids and *ankA* PCR amplicons were sequenced at The Ohio State University Comprehensive Cancer Center Nucleic Acid Shared Resource Facility.

Homologous gene sequences were identified using the Basic Local Alignment Search Tool (BLAST) algorithm BLASTn against the NCBI nucleotide database (15, 16). The sequences for *p44* and *ankA* were aligned and trimmed to the same PCR-amplified regions, and phylogenetic and evolutionary distance analysis was performed with Clustal Omega (17) in the MegAlign Pro program of Lasergene 17 (DNAStar, Madison, WI). To estimate confidence levels, bootstrap values for 1,000 replicates were calculated using the maximum likelihood method and the RAxML option of Lasergene 17. The sequence distance was calculated by dividing the number of nucleotide differences by the total number of nucleotides, with subsequent comparison among homologs.

### Indirect immunofluorescence assay

An indirect immunofluorescence assay (IFA) was performed using the *A. phagocytophilum* HZ strain as the antigen (10).

## RESULTS

### Horses diagnosed with EGA

**Fig 1:**
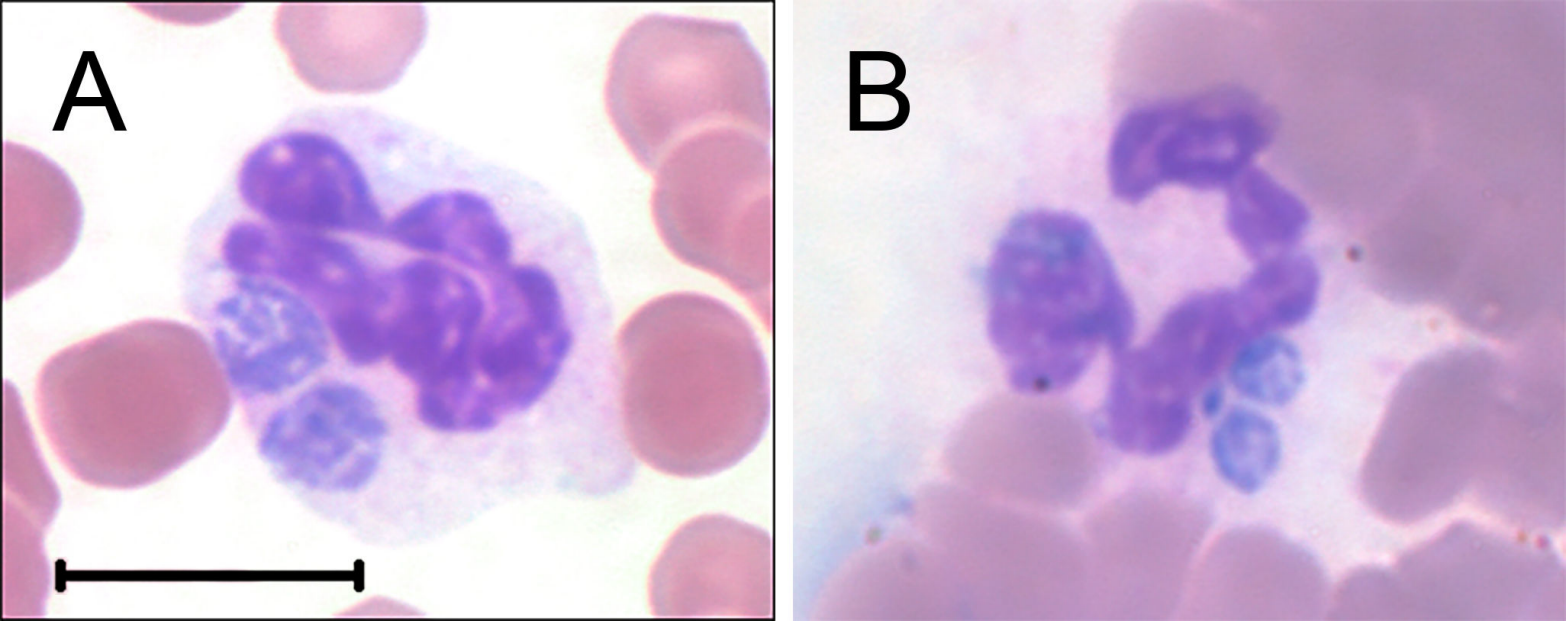
*A. phagocytophilum* inclusions in the cytoplasm of horse peripheral blood neutrophils. (**A**) BP18. (**B**) MK20. Hema 3 differential stain of buffy-coat smears. Bar: 10 µm.

Clinical signs of EGA and laboratory findings are summarized in Table 1. BP18 had fever, lethargy, inappetence, polysaccharide storage myopathy (a disorder characterized by muscle wasting, muscle soreness, and exercise intolerance), and marked thrombocytopenia. Examination of blood smears revealed a small percentage of neutrophils containing small (2–4 µm × 1–4 µm) fine-granular irregular-to-round-shaped blue-purple structures resembling *Anaplasma* inclusions, and a buffy-coat smear was prepared to confirm this observation (Fig. 1). MK20 had fever, lethargy, inappetence, a dental crown fracture with tooth root abscessation, a large mandibular draining tract, associated cellulitis with edema of the ventral mandible, nasal discharge, and thrombocytopenia. Examination of blood smears revealed that approximately 26% of the neutrophils contained morulae that were morphologically consistent with *A. phagocytophilum*, and a buffy-coat smear was prepared to confirm this observation (Fig. 1). GL21 had fever, lethargy, bilateral hindlimb cellulitis, right forelimb lameness, and thrombocytopenia. However, the blood smear was not examined. All three horses were treated with intravenous oxytetracycline and/or oral doxycycline and showed improvement within 1–2 days (Table 1).

**TABLE 1:** Clinical signs, clinical chemistry, and treatment outcome for three horses with HGA

Horse ID^*a*^	Sex^*b*^	Age	Sick (days)^*c*^	Depression	Anorexia	Fever	Lameness, ataxia	Laboratory data	IFA titer/PCR	Treatment outcome^*d*^
BP18	MC	17 yr	Few days	Yes	Yes	Yes	No	Marked thrombocytopenia, mild anisocytosis. *Anaplasma* morulae present in neutrophils.	Blood PCR positive	Full recovery
MK20	M	5 mo	Sick for months because of guttural pouch empyema	No	No	No	No	Moderate thrombocytopenia, mild hypochromic anemia, and mild lymphocytosis. *Anaplasma* morulae present in neutrophils.	*Anaplasma* IFA titer = 1:320 Blood PCR positive	Full recovery
GL21	F	16 yr	Fever due to hindlimb cellulitis	No	No	Yes	Yes, right forelimb	Thrombocytopenia, mild lymphopenia, elevated alkaline phosphatase, and hyperbilirubinemia.	Blood PCR positive	Full recovery

^*a*^ Horse as well as *A. phagocytophilum* strain ID. The last two numbers in each ID indicate the year of illness.

^*b*^ MC, male castrated; F, female; M, male.

^*c*^ The number of days the horse was observed to be sick by the owner before the attending veterinarian first examined the horse and collected blood samples.

^*d*^ All horses were treated with intravenous oxytetracycline and/or oral doxycycline.

### Analysis of *A. phagocytophilum ankA* sequences

**Fig 2:**
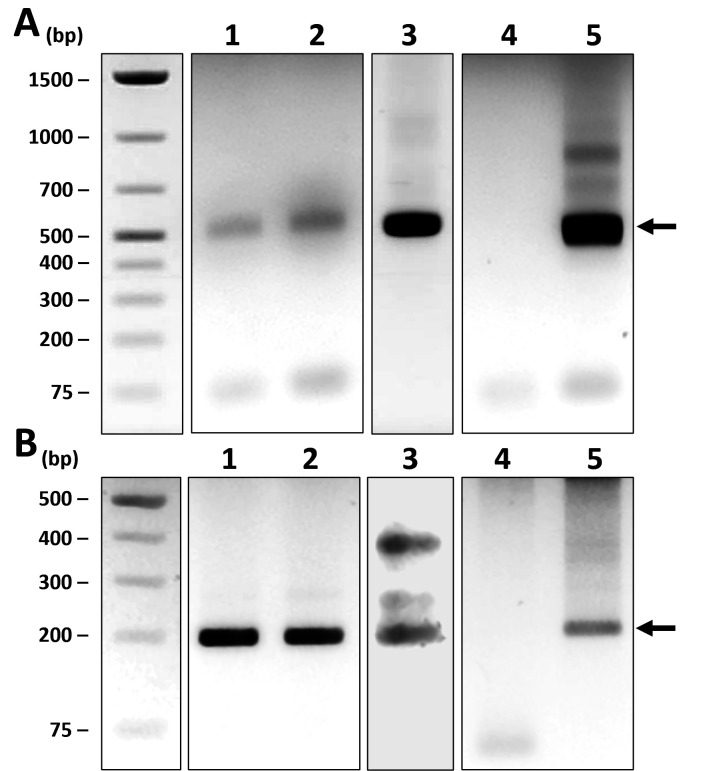
*A. phagocytophilum*-specific PCR. (**A**) *p44*-specific PCR. Lane 1, horse BP18; lane 2, horse MK20; lane 3, horse GL21; lane 4, nuclease-free water; lane 5, *A. phagocytophilum* HZ strain DNA. (**B**) *AnkA*-specific nested PCR. Lane 1, horse BP18; lane 2, horse MK20; lane 3, horse GL21; lane 4, nuclease-free water; lane 5, *A. phagocytophilum* HZ strain DNA. Horse-blood DNA was obtained from archived blood smear slides (BP18 and MK20) or a fresh blood sample (GL21).

**Fig 3:**
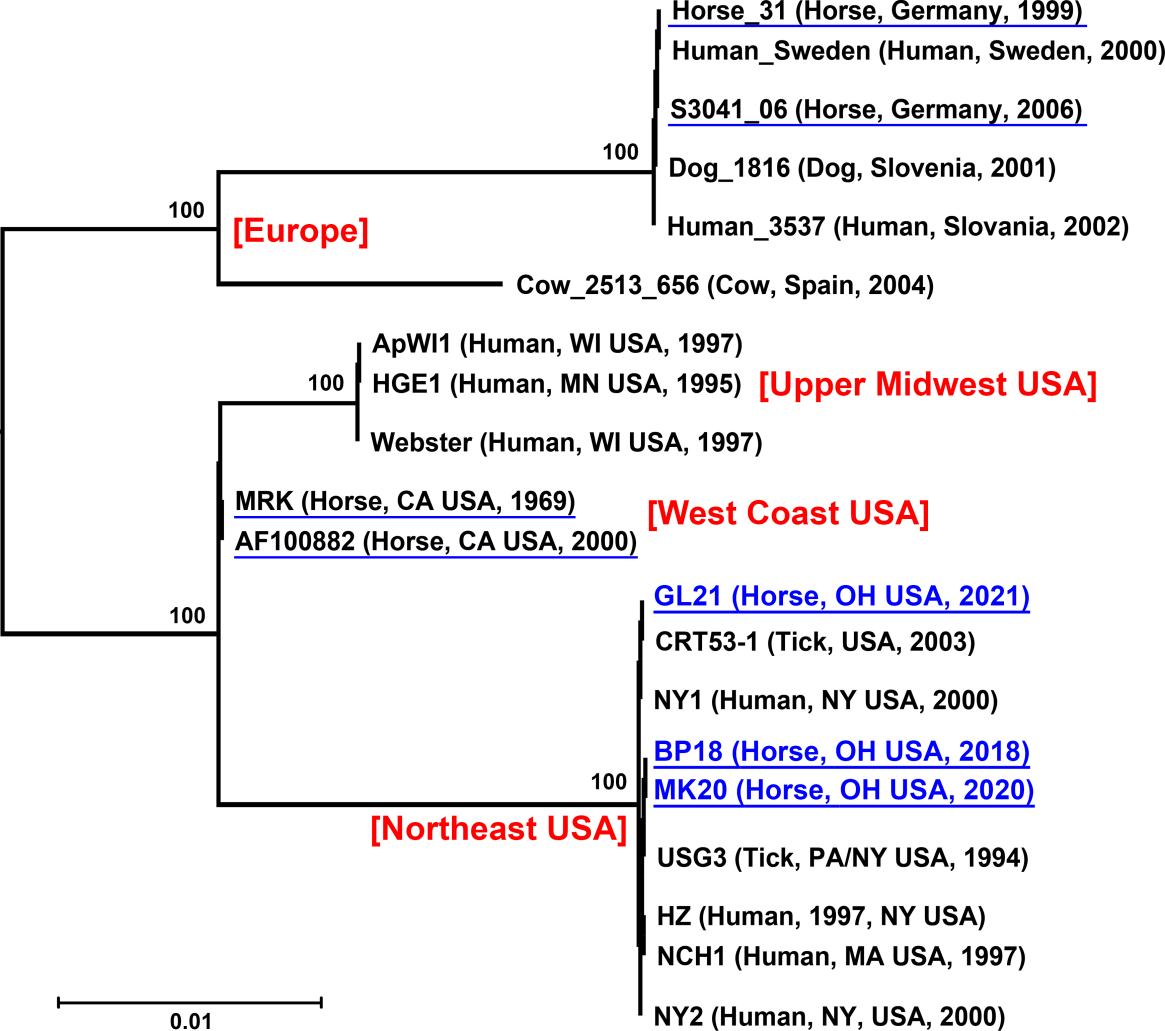
Phylogenetic tree for *A. phagocytophilum ankA* (199-bp nested PCR fragment). Sequences of *A. phagocytophilum ankA* corresponding to the 199-bp nested PCR region from humans, horses (shown with blue underlining), ticks, and other animals in the USA or Europe were aligned with sequenced PCR fragments amplified from the three horse clinical samples in our study (BP18, MK20, and GL21) using the Clustal Omega algorithm, and the phylogenetic tree was constructed using MegAlign Pro. Bootstrap values were calculated using the maximum likelihood method with the RAxML option. Numbers shown above each branch indicate bootstrap values. Scale bar: sequence distance. GenBank accession numbers for *ankA* genes: HZ, NC_007797/Locus_Tag: APH_RS03050; NY1, AF100883; NY2, AF100884; NCH1, LANT01000008/Locus_Tag: EPHNCH_1057; USG3, AF020521; Webster, GU236811; ApWI1, LAOF01000001/Locus_Tag: APHWI1_0236; HGE1, LASP01000004/Locus_Tag: APHDU1_1516; MRK, AF153716; CA horse, AF100882; CRT53-1, LAOD01000018/Locus_Tag: APHCRT_0858; Horse S3041_06, GU236863; Dog_1816, GU236812; Cow_2513_656, GU236729; Human_3537, GU236804; Human_Sweden, AF100888; Horse_31, AF482759.

The immunodominant AnkA protein of *A. phagocytophilum* is an effector protein secreted by the bacterial type IV secretion system into the host cytoplasm; there, AnkA regulates host cell signaling to facilitate bacterial infection (18, 19). *ankA*-specific PCR of DNA samples from all three horses revealed bands of the expected sizes (Fig. 2). Phylogenetic analysis of *ankA* sequences among *A. phagocytophilum* isolates from various geographic locations and host species revealed that they were highly divergent and could differentiate *A. phagocytophilum* strains (8). Therefore, we analyzed sequences of *A. phagocytophilum ankA* from humans, horses, ticks, and other animals in the USA and Europe and aligned these sequences with sequenced PCR fragments amplified from samples from the three horses in our study. Alignment of the 199-bp region of *ankA* and subsequent phylogenetic analysis revealed that *ankA* fragments from the three horse samples were completely identical to the HZ strain (human isolate from the state of New York) (19) but were distinct from those of horses, ticks, or other animal isolates from California or Europe (Fig. 3). Furthermore, they could be clustered based mainly on the geographic origin of the *A. phagocytophilum* isolates regardless of host animal species or isolation year (Fig. 3).

### Analysis of *p44* sequences

**Fig 4:**
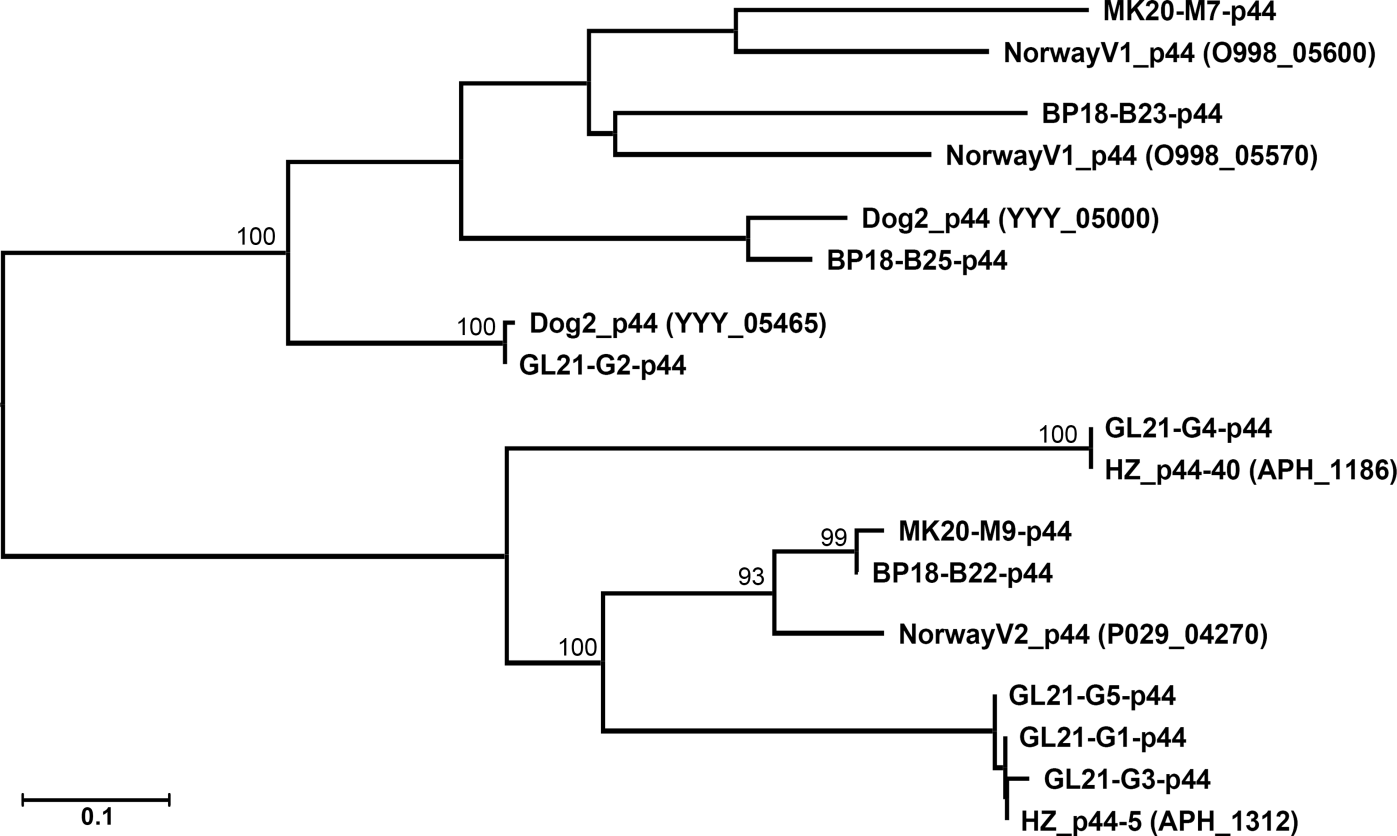
Phylogenetic tree for *A. phagocytophilum p44* genes. Fragments of *A. phagocytophilum p44* genes were PCR amplified from horse samples (BP18, MK20, and GL21) and cloned into plasmid pCR-Blunt II-TOPO. Sequences were determined and aligned with the best-matched *p44* genes of the HZ strain (human isolate) using the Clustal Omega algorithm, and the phylogenetic tree was constructed using MegAlign Pro. Bootstrap values for 1,000 replicates were calculated using the maximum likelihood method with the RAxML option. Numbers shown above each branch indicate bootstrap values. Scale bar: sequence distance.

P44/Msp2 proteins are major surface antigens that are useful for serodiagnosis (20, 21). The *A. phagocytophilum* HZ genome encodes 113 copies of full-length and truncated *p44* genes belonging to the OMP-1/MSP2/P44 superfamily (22). Each of these *p44* (*msp2*) genes has a central hypervariable region flanked by 5′ and/or 3′ conserved sequences. Diverse *p44* paralogs are expressed at a single locus owing to a unidirectional gene conversion (recombination) event from the 113 donor *p44* loci of *A. phagocytophilum* in mammals and ticks, and these paralogs have facilitated adaptation to the host environment (23 – 26). By designing primers targeting the conserved 5′ and 3′ ends (14), the *p44* gene fragments could be amplified from the three horse DNA samples with subsequent cloning into plasmid pCR-Blunt II-TOPO and sequencing. NCBI BLASTn searches revealed that the five sequences within the hypervariable region of each *p44* gene in samples taken from horses BP18 and MK20 did not match exactly to any sequence in the NCBI GenBank nucleotide database (in total, 76,024,418 *A*. *phagocytophilum* nucleotide sequences; Table S2). One of the five sequences of the hypervariable region of *p44* in samples taken from horse GL21 was identical to one of the Minnesota Dog2 *p44/msp2* sequences that share 100% of its *p44/msp2* repertoire with the human strain HGE1 from Minnesota (9) (Fig. 4). The remaining four of five sequences of the hypervariable region of *p44* from horse GL21 were similar or identical to those of the full-length or truncated genes (*p44-5, p44-40*) from *A. phagocytophilum* HZ (Table S2). The sequence variation of *p44-5* likely reflects a mixed population of bacteria. *A. phagocytophilum* HZ was not handled in the laboratory during the *p44* PCR and sequencing experiments; however, because these sequences were similar to those from *A. phagocytophilum* HZ from an HGA patient and cultured in the human promyelocytic leukemia cell line HL-60, human *HPRT*-specific PCR was performed with GL21 DNA. The PCR results demonstrated that the GL21 specimen was not contaminated with human DNA, excluding the possibility of *A. phagocytophilum* HZ contamination. Phylogenetic analyses with *p44* genes of *A. phagocytophilum* revealed that all sequences obtained from samples of BP18 and MK20 were unique and distinct from those of GL21 or *A. phagocytophilum* HZ (Fig. 4).

### Geographic distribution of EGA cases in Ohio based on IFA

**Fig 5:**
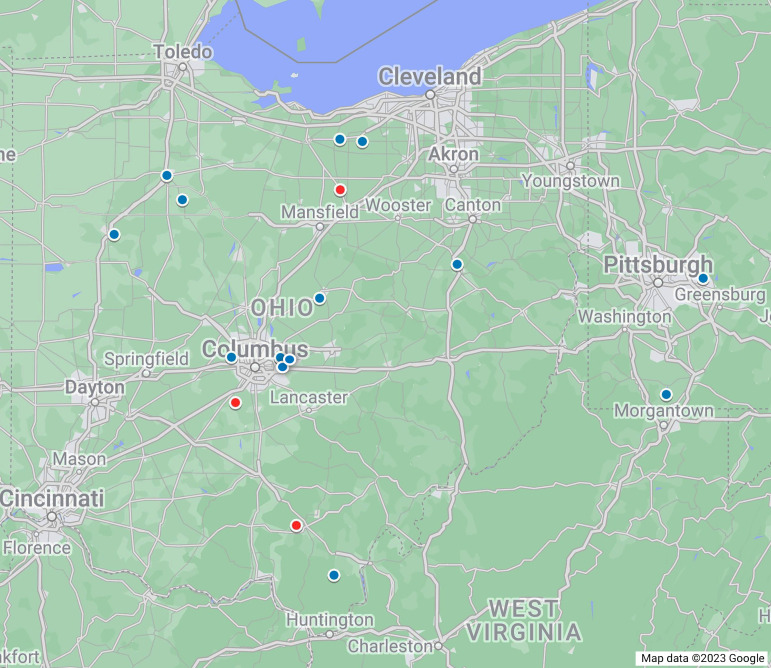
Geographic locations of the horses with EGA in Ohio and nearby Pennsylvania from 2018 to 2023. Positive cases were identified by IFA (blue dots) and PCR (red dots).

From 2018 to 2023, 14 cases with clinical signs consistent with EGA were diagnosed by IFA in Ohio and nearby Pennsylvania, suggesting that the PCR and sequence-confirmed EGA cases in this study (BP18, MK20, and GL21) are not isolated events but represent the tip of the iceberg of the under-characterized EGA cases in Ohio and nearby counties in Pennsylvania (Fig. 5).

## DISCUSSION

We report the presence of EGA in Ohio based on results obtained from PCR and *A. phagocytophilum*-specific gene sequencing. The results will help raise awareness of the potential threat of tick-borne diseases, particularly EGA in Ohio. The primary mammalian reservoir of *A. phagocytophilum* is the white-footed mouse (*Peromyscus leucopus*), and *Ixodes scapularis* ticks are the biological vectors in the eastern USA (27). These ticks are also the reservoir and vector for the bacterium *Borrelia burgdorferi*, the agent of Lyme disease, and previous studies reported increasing numbers of *I. scapularis* ticks and *B. burgdorferi* infection of ticks and dogs in Ohio (28).

Clinical signs of EGA are fever lasting 1–9 days, depression, moderate body wasting, limb edema, petechiae, icterus, ataxia, and reluctance to move [reviewed in references (7, 29 – 31)]. Opportunistic infections may also occur. The disease is inapparent to mild and is usually not fatal except for serious physical injury resulting from ataxia or secondary infections (32). Changes in complete blood cell count or blood chemistry typically involve mild to severe thrombocytopenia, anemia, leukopenia due to lymphopenia and neutropenia, and hyperbilirubinemia (32). Because the clinical signs of EGA can vary greatly, laboratory diagnosis is required. Similar to HGA, EGA can be diagnosed by examination of a blood smear stained by Romanowsky stains such as Wright-Giemsa, PCR based on 16S rDNA, *p44*, or other genes of *A. phagocytophilum*, and/or serologic tests using *A. phagocytophilum* cultured in HL-60 cells or P44 proteins or peptides as the antigen (7). Culture isolation of *A. phagocytophilum* from the blood of infected horses is rarely performed (33), although *A. phagocytophilum* has been isolated from blood samples from several human patients with HGA (11, 34, 35). The present study demonstrates a convenient means of retrospective molecular diagnosis of EGA using archived blood smears, especially when fresh or frozen blood samples are not available.

Previous studies have shown that the phylogeny of *A. phagocytophilum ankA* genes from various isolates correlates partially with the geographic regions of *Anaplasma* isolates (36) and/or their host species (37). Our phylogenetic analysis of the variable regions of *ankA* genes (200 bp) suggested that the three *A. phagocytophilum* strains from horses in Ohio were related to *A. phagocytophilum* isolates from the northeastern USA regardless of host species. This is in agreement with previous studies based on whole genome sequence comparison showing that the Dog2 strain of *A. phagocytophilum* from Minnesota is closely related to the HGE1 human strain from Minnesota (9).

As many *p44* genes are present in the genome of *A. phagocytophilum,* sequences of a small number of *p44* are not useful for taxonomic analysis of *A. phagocytophilum* strains. However, *p44* sequences provide an additional PCR testing target to validate the EGA diagnosis and may also inform P44 antigenic diversity/variation of *A. phagocytophilum* strains present in the blood from horses with clinical EGA. Ideally, sequencing of the whole genome (9) would allow more precise analysis, as short *p44* sequences may align even with other *Anaplasma* species, such as *Anaplasma* sp. SA dog and *A. platys* in dogs (38). However, in most clinical diagnostic blood samples, this is not possible, as they contain only a small amount of *Anaplasma* DNA (unless culture isolated), or DNA was severely fragmented due to fixation and staining of blood smears on slides and storage at ambient temperature. Based on a comparison of hypervariable region sequences of *p44*, our results affirmed *Anaplasma* infection and the presence of genes encoding diverse P44 antigens among *A. phagocytophilum* strains infecting horses in Ohio.

Although 16S rDNA sequences are relatively conserved, *A. phagocytophilum* detected in horses from Connecticut and Minnesota had 16S rDNA variable region sequences similar to those of *A. phagocytophilum* isolated from humans (39, 40). These findings are in agreement with previous studies showing that *A. phagocytophilum* blood culture isolates from humans can infect horses and cause clinical diseases similar to naturally occurring EGA in horses (41 – 43). Thus, awareness of EGA is not only important for veterinary medicine but also relevant to public health because horses with EGA may serve as sentinels for HGA. To our knowledge, however, natural transmission of *A. phagocytophilum* from infected horses to humans, or vice versa, has not been reported.

Serological tests are generally specific to a certain bacterial species and cannot be used to distinguish individual strains. *A. phagocytophilum* strains vary slightly in nucleotide sequences, with 99 ± 5% identity (up to 5 bp) for 16S rDNA and 99 ± 0% identity for the chaperonin gene *groESL*. The sequences of *ankA* vary substantially between strains (8, 34). These and potentially other genes, such as those previously described (9), allow for more detailed comparison among strains. For example, diverse *ankA* sequences are found in naturally infected ticks and wild deer, including *ankA* sequences that have not been detected in humans or domesticated animals (37, 44). Further, the use of multi-locus sequence typing (MLST) would be beneficial for future studies to detect *A. phagocytophilum* strains/variants (45). Future molecular studies of *A. phagocytophilum* variants will shed light on the evolution, population dynamics, and ecology of naturally occurring *A. phagocytophilum* strains and will help identify risks for outbreaks of zoonosis and veterinary diseases.

*A. phagocytophilum* DNA has been detected in several species of *Ixodes* ticks (*I. scapularis*, *I. pacificus*, *I. spinipalpis*, *I. ricinus*, *I. persulcatus*, and *I. ovatus*) in the USA, Europe, and Asia (27, 46). Naturally infected ticks can transmit *A. phagocytophilum* to naïve mammals (47 – 49). Once ticks acquire the bacterium from infected mammals through a blood meal, the bacterium is maintained from the larva or nymph stage to the adult stages of metamorphosis and is transmitted to mammals during the next blood meal (27, 42, 50). Because evidence is lacking for transovarial transmission (i.e., from adult ticks to eggs), larvae cannot transmit the bacterium to mammals, yet infected nymphs and adult ticks can transmit the bacterium. Although *Ixodes* ticks often feed on white-tailed deer, in the USA, the deer are infected with *A. phagocytophilum* strain Variant 1 rather than with the human strain (51). Diverse *A. phagocytophilum* strains are also found in animals and ticks in Japan and Russia (52 – 54), where HGA and EGA are rarely reported. These findings imply that the zoonotic potential of *A. phagocytophilum* depends on not only the transmissibility, habitats, and population density of ticks and infected mammals but also the genetic variants of *A. phagocytophilum*. In 1969, Gribble suggested that horses may be accidental hosts based on the low incidence of EGA (30). Which strains infect and cause EGA remain to be studied further (27).

Intravenous administration of oxytetracycline at 7 mg/kg body weight is reported to be effective for the treatment of *A. phagocytophilum* infection (31). The three EGA PCR-positive horses in our study were treated with intravenous tetracyclines and/or oral doxycycline and recovered uneventfully. Because *A. phagocytophilum* is most likely transmitted by *Ixodes* sp. ticks, tick repellents and insecticides are therefore expected to be effective preventative measures, as currently no vaccine has been developed against *A. phagocytophilum*.

## Data Availability

The partial sequences of *ankA* and *p44* from horses BP18, MK20, and GL21 were deposited into and are publicly available at NCBI GenBank (https://www.ncbi.nlm.nih.gov/genbank/) with accession numbers from OQ927948 to OQ927960.

## SUPPLEMENTAL MATERIAL

Supplemental material spectrum.02632-23-s0001.docx Tables S1 and S2.

ASM does not own the copyrights to Supplemental Material that may be linked to, or accessed through, an article. The authors have granted ASM a non-exclusive, world-wide license to publish the Supplemental Material files. Please contact the corresponding author directly for reuse.
