# Supplementary material for: Strains of Anaplasma phagocytophilum from horses in Ohio are related to isolates from humans in the northeastern USA
Source: Microbiol Spectr. 2023 Oct 26;11(6):e02632-23. doi: 10.1128/spectrum.02632-23 (PMC10715102; doi:10.1128/spectrum.02632-23)
Supplement: Supplemental material — Tables S1 and S2. [file spectrum.02632-23-s0001.docx]

**Table S1. Primer sequences for *A. phagocytophilum* *p44* and *ankA* and for human *HPRT***

| **Target gene** | **Primer name** | **Direction** | **Sequence (5'–3')** | **References** |
| --- | --- | --- | --- | --- |
| *Anaplasma*  *phagocytophilum p44* | p3708 | Forward | GCTAAGGAGTTAGCTTATGAT | (1) |
|  | p4257 | Reverse | AAGAAGATCATAACAAGCATT | (1) |
| *Anaplasma*  *phagocytophilum ankA* | AnkA-F | Forward | AGACAACTTTGACCGCTGAAGCAC | this study |
|  | AnkA-R | Reverse | GCAGCTGCTATATGTAAAGCCGTC | this study |
|  | AnkA-F2 | Forward | GCATTTAAAGCGTGTTGTAGAAGC | this study |
|  | AnkA-R2 | Reverse | TGCTAAATGCAAGGCTGGAG | this study |
| Human *HPRT* | HPRT1P3-F | Forward | ATTGGCTCTATCATAGGAATGGCTC | this study |
|  | HPRT1P3-R | Reverse | CATGGACTGATTGTGAACAGGACC | this study |

1. Zhi N, Ohashi N, Rikihisa Y. 1999. Multiple *p44* genes encoding major outer membrane proteins are expressed in the human granulocytic ehrlichiosis agent. J Biol Chem 274:17828-17836.

**Table S2. Best matches of sequenced *p44* from horses in other *A. phagocytophilum* strains**

| **Horse ID** | ***p44* clone ID (length, bp)** | **Best Matched *p44* gene (Strain / host species;  GenBank Accession # / Locus tag; Gene length)^1^** | **% Identity  (matched region)^1^** |
| --- | --- | --- | --- |
| **BP18** | B22 (501) | *p44/msp2* family outer membrane protein, silent (Norway Variant2 / sheep; CP015376 / P029_04270; 473 bp*)* | 89% (1 ~ 470) |
|  | B23 (513) | *p44/msp2* family outer membrane protein  (Norway Variant1 / sheep; CP046639 / O998_05570; 1,269 bp) | 76% (338 ~ 837) |
|  | B25 (510) | *p44/msp2* family outer membrane protein, silent  (Dog2 / dog; CP006618 / YYY_05000; 719 bp) | 90% (1 ~ 513) |
| **MK20** | M7 (505) | *p44/msp2* family outer membrane protein  (Norway Variant1 / sheep; CP046639 / O998_05600; 1,101 bp) | 78% (85 ~ 588) |
|  | M9 (501) | *p44/msp2* family outer membrane protein, silent (Norway Variant2 / sheep; CP015376 / P029_04270; 473 bp) | 88% (1 ~ 473) |
| **GL21** | G1 (504) | *p44-5* outer membrane protein, silent  (HZ / human; CP000235 / APH_1312; 876 bp) | 100% (16 ~ 561) |
|  | G2 (516) | *p44/msp2* family outer membrane protein, silent  (Dog2 / dog; CP006618 / YYY_05465; 1,101 bp) | 99% (118 ~ 633) |
|  | G3 (504) | *p44-5* outer membrane protein, silent (HZ / human; CP000235 / APH_1312; 876 bp) | 98% (16 ~ 561) |
|  | G4 (477) | *p44-40* outer membrane protein, silent  (HZ / human; CP000235 / APH_1186; 783 bp) | 100% (82 ~ 600) |
|  | G5 (504) | *p44-5* outer membrane protein, silent  (HZ / human; CP000235 / APH_1312; 876 bp) | 99% (16 ~ 561) |

1. Fragments of *A. phagocytophilum p44* genes were PCR amplified from horse clinical samples (BP18, MK20, and GL21), and cloned into pCR-Blunt II-TOPO plasmid. Sequences were determined and *p44* genes (primer sequences removed) of PCR fragments were searched against NCBI non-redundant nucleotide database (8,345,909 sequences total, organism limited to *A. phagocytophilum*) by Blastn algorithm, and the best matched *p44* genes with lowest E-value for each clone were identified.
